# Editorial: Asian health sector growth in the next decade—Optimism despite challenges ahead

**DOI:** 10.3389/fpubh.2023.1150917

**Published:** 2023-02-20

**Authors:** Mihajlo Jakovljevic, Liang Wang, Chiranjivi Adhikari

**Affiliations:** ^1^Institute of Advanced Manufacturing Technologies, Peter the Great St. Petersburg Polytechnic University, Saint Petersburg, Russia; ^2^Institute of Comparative Economic Studies, Hosei University, Tokyo, Japan; ^3^Department of Global Health Economics and Policy, University of Kragujevac, Kragujevac, Serbia; ^4^Department of Public Health, Robbins College of Health and Human Sciences, Baylor University Waco, TX, United States; ^5^Indian Institute of Public Health Gandhinagar (IIPHG), Gandhinagar, India; ^6^Department of Public Health, School of Health and Allied Sciences (SHAS), Pokhara University, Pokhara, Nepal

**Keywords:** Asia-Pacific, China, Japan, universal health coverage (UHC), health expenditure, developing countries, health care cost, medical care

The Western Pacific and the Southeast WHO regions of Asia have a variety of economies, from middle-income India, Laos, and Myanmar to high-income OECD countries, such as Japan and South Korea. These regions are home to a sizable population and act as an epicenter of global manufacturing and industrial capacity (1).

While most of Asia was under colonial rule before World War II, it became the fastest-growing economic region of the world in recent decades, manifesting some of the world's longest economic booms, starting from Japan in the 1950s, to South Korea, China, and recently to India and the Tiger Cub Economies, such as Singapore and Malaysia (2). In contrast, these boomed economies later faced setbacks due to various reasons, such as the 1997 Asian financial crisis, which spread to the ASEAN (Association of South East Asian Nations) region (3), the contraction of the GDP in countries such as Indonesia and Sri Lanka, the aftermath of the heavy inflow of foreign aid that was demanded following a tsunami and other disastrous natural calamities (4), the impact of the 2008–2009 global recession and its effects in Asian countries (5), and the recent COVID-19 pandemic. These caused multi-faceted economic effects in Central Asia (6), and other Asian countries.

In addition to these ups and downs, a meager proportion (approximately 4–7%) of total GDP has been invested in the health sector in most Asian countries, in comparison to approximately 12% or more of that in European countries, the United States, and other high-income countries. Increased health awareness leading to increased health demand has, directly and indirectly, pressurized the ruling governments in the region to invest in the health sector, leading to multiple and mixed avenues of different opportunities and challenges for health sector growth.

Of the total 17 manuscripts accepted for this Research Topic, 14 are original research papers, two are reviews, and one is a perspective article. The manuscripts and total numbers are found under the following six themes: Primary Health Care (PHC) and Health Systems Development (HSD) (four manuscripts); (Health) insurance, governance, and universal health coverage (UHC) (four manuscripts); public health screening (two manuscripts); urban and green economics, and sustainability (two manuscripts); health care profession, ethics, and leadership (three manuscripts); and health tourism and migrants' health (two manuscripts). Geographic coverage is as follows: seven and two manuscripts are from China and Nepal, respectively, and one each represents Asia, the Middle East and North Africa (MENA), Mongolia, Malaysia, South Korea, Japan, and Indonesia (Table 1).

**Table 1:** Scopes and summary findings of the studies.

**References**	**Title**	**Country/Region**	**Type of article**	**Major findings/conclusions**
**Scope/Areas/Theme 1: Primary health care (PHC) and health systems development (HSD)**				
1. Katoue et al.	Healthcare system development in the middle east and North Africa region: challenges, endeavors, and prospective opportunities	Middle East and North Africa (MENA)	Review	• Explored the experiences, challenges, and future opportunities of various governments' initiatives for health system development (HSD).
2. Poudel et al.	Disempowered mothers have undernourished children: how strong is the intrinsic agency?	Nepal	Original research	• Maternal empowerment, basically an intrinsic agent, strongly affects children's nutritional status, especially child wasting and being underweight.
3. Wenang et al.	Availability and accessibility of primary care for remote, rural, and poor populations in Indonesia	Indonesia	Original research	• Guiding referral and utilization of primary care will be a key success factor for effective and efficient usage of available healthcare infrastructure and achievement of universal health coverage.
4. Jackojevic et al.	Governmental investments in hospital infrastructure among regions and its efficiency in China: an assessment of building construction	China	Original research	• Investment efficiency was higher when switching governmental investment predisposition in the aspect of healthcare infrastructure construction toward less developed regions.
**Scope/Areas/Theme 2: (Health) insurance, governance, and UHC**				
5. Paneru et al.	Adopting social health insurance in Nepal: A mixed study	Nepal	Original research	• Further explored that social health insurance may not suffice, and so, future perspectives of HI may be taken up with alternate strategies; •Prediction models were presented.
6. Li et al.	Official tenure and governance effectiveness of China's basic pension insurance system: An inverted u-shaped curve	China (31 Provinces)	Original research	• Localized government officials, rather than non-localized ones, showed positive governance efficiency.
7. Chung	Characteristics associated with financial or non-financial barriers to healthcare in a universal health insurance system: A longitudinal analysis of Korea's health panel survey data	South Korea	Original research	• Along with financial barriers to be addressed, current universal health insurance systems need targeted policy instruments to minimize non-financial barriers to healthcare to ensure effective universal health coverage.
8. Tian et al.	Institutional design and incentives for migrant workers to participate in social insurance in China: evidence from a policy experiment in Chengdu city	China	Original research	• Changing the social insurance model of migrant workers from comprehensive social insurance to urban employee insurance reduces the incentives for migrant workers to participate in insurance and harms the overall welfare of migrant workers. •Policy experiment using the difference-in-differences model.
**Scope/Areas/Theme 3: Public health screening**				
9. Wang et al.	Benefit-to-harm ratio and cost-effectiveness of government-recommended gastric cancer screening in China: a modeling study	China	Original research	• The gastric cancer risk score scale (GCRSS) strategy is effective and cost-effective in reducing the gastric cancer disease burden and the optimal strategy would occur from the age of 40; •Microsimulation model and deterministic and probabilistic sensitivity analyses were carried out.
10. Shah et al.	Cost-effectiveness of portable electrocardiogram for screening cardiovascular diseases at primary health centres in Ahmedabad District, India	India	Original research	• Portable electro-cardiograms to screen cardiac abnormality at the PHC level is highly cost-effective for high-risk adult and symptomatic cases; •Use of a decision-analytic model.
11. Chen et al.	Analysis of spatiotemporal characteristics of urban economic resilience and influencing factors in the Guangdong-Hong Kong-Macao Greater Bay Area	China (Guangdong-Hong Kong-Macao Greater Bay Area)	Systematic Review	• Economic resilience increased in the middle and south regions and decreased in the northwest, and economic status and economic response were the main dimensions affecting resilience •A Pressure-State-Response (PSR) model was used in the post-pandemic situation.
12. Shao et al.	How fast are Asian countries progressing toward a Green Economy? Implications for public health	Asia	Original research	• Some countries have reached a high green development level, and the medium-income ones move fast toward a green economy, while some low-income countries get worse.
**Scope/Areas/Theme 5: Health care profession, ethics, and leadership**				
13. Wang et al.	Sustainability of nursing leadership and its contributing factors in a developing economy: A study in Mongolia	Mongolia	Original research	• Behavior and problem-solving skills positively contribute to nursing leadership. Transformational ability also contributed significantly to nursing leadership.
14. Dong et al.	Satisfaction as a mediator and its interaction with adherence to labor analgesia protocols: A cross-sectional survey of Chinese medical personnel	China	Original research	• Medical personnel working at PHC levels should be considered for comprehensive incentives, including training for them as well as staff to improve the use of labor analgesia.
15. Ozaki et al.	How do institutional conflicts of interest between pharmaceutical companies and the healthcare sector become corrupt? A case study of scholarship donations between the Department of Anesthesiology, Mie University, and Ono Pharmaceutical in Japan	Japan	Perspective article	• Highlighted potential institutional remedies that may alleviate ICOIs and corrupt behavior affecting the healthcare sector.
**Scope/Areas/Theme 6: Health tourism and migrant health**				
16. Fenming et al.	Investigating revisit intention of medical tourists in China through nutritional knowledge, perceived medical quality, and trust in the physiologist: A recommendation on health tourism policy measures	China	Original research	• Nutritional knowledge, perceived medical quality, and trust in physiologists significantly influence the revisit intention of medical tourists; •Structural equation modeling using Smart PLS.
17. Li et al.	Measuring Patients' Satisfaction toward Health Tourism in Malaysia through hospital environment, nutritional advice, and perceived value: A study on Chinese exchange	Malaysia	Original research	• Hospital environment, nutritional advice, and perceived value significantly influence patients' satisfaction; •Structural equation modeling using Smart PLS.

The editors observed that shifting infrastructure development to less developed regions, focusing on women's empowerment, and guiding the referral system may enhance access to PHC and thereby advance health and social development (HSD); for which the non-state actors should work in close collaboration with the state. In order to achieve UHC, the health sector in the next decade is expected to grow and move beyond HSD. For this, in addition to financial and non-financial barriers to health insurance, alternative strategies to existing insurance schemes are crucial, including social health insurance (HI), as some Asian and African countries are moving closer to UHC by HI reforms (7, 8); improved governance through localized employees; public health screening with cost-effective tools and strategies, such as portable ECGs and the gastric cancer risk score scale (GCRSS), which can be further captivated with the Health Technology Assessment (HTA) (9) and may further help in reducing the ongoing catastrophic expenditures of households with noncommunicable diseases (NCDs) (10); and the leadership skills and training of health care professionals. Another area of growth in the health sector is health tourism, for which patient satisfaction and enhancing the trust between the health professionals and the patient are warranted. Since domestic manufacturing is not meeting the ever-rising demand for medical services, which is visible in terms of more significant health and pharmaceutical expenditures (11) on equipment and medicines (12), cross-country health and medical tourism is inevitable. To meet this demand and intra-country unmet healthcare challenges in some countries while also seizing other markets, institutions should consider other informed solutions to solve conflicting interests between the pharma company and the health sector. Furthermore, economic resilience after the COVID-19 pandemic and maintaining the pro-green economies may contribute to the region's health sector growth and to its sustainability, including the ability to face challenges to meet sustainable development goals (13).

## Conclusive remarks

The editors believe these valuable and diverse topic contributions might open new horizons of knowledge. Last but not least this is a unique opportunity to open the floor for a public debate on the Asian challenges through the lens of the Global South's health sector growth (12). The main goal of this Research Topic was to describe the progress made in the healthcare arena in Asian Emerging economies. A diverse group of authors coming from academia, industry, governing authorities, and professional associations attempted to provide a thorough overview of the status of the healthcare sector growth in Asian-Pacific economies (14). We hope that this collection of articles can trigger curiosity among aspiring Asian public health authorities, HTA experts, clinical physicians, economists, and public communities alike.

## Author contributions

MJ and CA prepared the manuscript draft while MJ, CA, and LW revised it for important intellectual content. All authors fulfill ICMJE conditions for full authorship. All authors contributed to the article and approved the submitted version.
